# Functional genetic potential of benthic microbial mat communities in Arctic, Antarctic, and sub-Antarctic lakes

**DOI:** 10.1093/femsec/fiag060

**Published:** 2026-06-13

**Authors:** Jill De Visscher, Bjorn Tytgat, Dominic A Hodgson, Annick Wilmotte, Anne Willems, Elie Verleyen, Wim Vyverman

**Affiliations:** Laboratory of Protistology and Aquatic Ecology, Ghent University, Krijgslaan 297-S8, 9000 Ghent, Belgium; Laboratory of Protistology and Aquatic Ecology, Ghent University, Krijgslaan 297-S8, 9000 Ghent, Belgium; British Antarctic Survey, Natural Environment Research Council, High Cross, Madingley Road, Cambridge CB3 0ET, United Kingdom; InBios-Molecular Diversity and Ecology of Cyanobacteria, University of Liège, Allée du Six Août 11, B6a, Quartier Agora, 4000 Liège, Belgium; Laboratory of Microbiology, Ghent University, K.L. Ledeganckstraat 35, 9000 Ghent, Belgium; Laboratory of Protistology and Aquatic Ecology, Ghent University, Krijgslaan 297-S8, 9000 Ghent, Belgium; Laboratory of Protistology and Aquatic Ecology, Ghent University, Krijgslaan 297-S8, 9000 Ghent, Belgium

**Keywords:** shotgun sequencing, metagenomics, cold adaptation, climate, macroecology, lakes

## Abstract

Benthic microbial mat communities are key drivers of ecosystem functioning in polar lakes and ponds, forming the base of aquatic food webs and contributing substantially to nutrient cycling. Although Arctic, sub-Antarctic, and Antarctic microbial mats differ in community composition, their functional genetic potential remains poorly understood. We applied shotgun metagenomic sequencing to study 17 microbial mat communities from Arctic and (sub-)Antarctic lakes differing in salinity, catchment vegetation, and climatic conditions. Stress response genes, especially cold stress, and phosphorus cycling and metabolism genes were highly abundant in all lakes. A large proportion of functional genes was shared between regions, with core functions dominated by transport mechanisms and energy production. However, clear differences in particular gene abundances were observed. Several East-Antarctic lakes and inland ponds in the Transantarctic Mountains showed a dominance of oxygenic photosynthesis and Calvin cycle genes for carbon fixation, likely reflecting the dominance of Cyanobacteriota. In Arctic and sub-Antarctic lakes with catchment vegetation and higher arthropod abundances, lignin and chitin degradation genes were more important. Our study shows that, despite distinct biogeographic patterns in community composition, the functional genetic potential of polar lake microbial mats mainly reflects climatic and local environmental conditions, emphasizing specific adaptations to extreme polar environments.

## Teaser

Functional characterization of polar benthic microbial mat communities showing the important influence of climatic and local environmental conditions on community functioning, highlighting the adaptation of these communities to extreme environments. Photographs of lakes courtesy of Elie Verleyen and Dominic Hodgson.

## Introduction

Polar lakes are hotspots of primary production and support a disproportionate amount of inland biodiversity compared with the often barren or poorly vegetated surrounding cold desert soils in the High-Arctic and Antarctica (Hawes et al. 2023, Tytgat et al. 2023). Polar regions further harbour a large diversity of different lake ecosystems, ranging from freshwater to hypersaline, from perennially ice-free to permanently ice-covered, and permanently or seasonally stratified (Verleyen et al. 2011, Vincent and Laybourn-Parry 2008). A general characteristic of polar lakes, and lakes in general, is that microorganisms typically form the base of the food web (Ellis-Evans 1996, Cavicchioli 2015). To survive in the cold polar conditions, with low nutrient concentrations and seasonally highly variable light levels, microorganisms might adopt several strategies, including a mixotrophic lifestyle, as well as cellular modifications, such as changes in the lipid composition of cell membranes, cold-adaptation of catalytic enzymes, and production of proteins necessary for stabilization and correct folding of DNA and mRNA (Rodrigues and Tiedje 2008, Vincent and Laybourn-Parry 2008, Collins and Feller 2023). The generally oligotrophic conditions in polar lakes sustain only low phytoplankton densities in their transparent water columns (Lizotte 2008). Consequently, a conspicuous feature of many polar lakes and ponds is their extensive benthic microbial mats (Sabbe et al. 2004), Vincent et al. 1993), which are structurally complex layered communities, typically perennial, covering the bottom of these aquatic systems (Wharton et al. 1983). The capability of biota in these microbial mats to scavenge nutrients from the water column and the underlying sediments, combined with their efficient recycling of nutrients within the mat itself (Vincent et al. 1993, Varin et al. 2010) result in primary production in the mats often exceeding that in the water column (Vincent et al. 1993, Ellis-Evans 1996). Nutrient concentrations can be one to two orders of magnitude higher in Antarctic cyanobacterial mats compared to the water column, with particularly dissolved reactive phosphorus and ammonium concentrations higher in the mats (Vincent et al. 1993). Furthermore, the Cyanobacteriota structuring these mats produce exopolysaccharides (EPS), which allows bacterial aggregate formation (Varin et al. 2012). This, together with the low grazing pressure, which is particularly the case in Antarctic lakes, can result in the build-up of high biomass stocks (Ellis-Evans 1996).

Our understanding of the taxonomic composition and diversity of these microbial mats has greatly advanced in recent years due to the application of high-throughput DNA sequencing techniques. Although microbial mats and biofilms are generally dominated by Cyanobacteriota and Pseudomonadota, their food webs are clearly different between Arctic and (sub-)Antarctic lakes (Varin et al. 2012, Kleinteich et al. 2017, Tytgat et al. 2023). Antarctic food webs are more simplified and truncated (Vincent and Laybourn-Parry 2008), and mats are generally dominated by cyanobacteria and chlorophytes, with ciliates, tardigrades, and rotifers as the main grazers. By contrast, Arctic lakes typically have a more complex food web structure due to a higher species diversity and abundance of metazoa (Vincent and Laybourn-Parry 2008) and their mats are additionally characterized by (other groups of) Cyanobacteriota, Pseudomonadota, and Ochrophyta (Varin et al. 2012, Tytgat et al. 2023). In addition to in-lake primary producers, Arctic and sub-Antarctic, and some Maritime Antarctic, lakes generally have vegetation, namely algae, mosses, and angiosperms, in their catchments, thus providing an allochthonous source of organic matter. By contrast, the organic matter in Antarctic lakes is often derived from autochthonous productivity. These differences stem from different environmental and climatic conditions between the Arctic, sub-Antarctic, and Antarctic lakes, but are also due to the different evolutionary histories of the regions (Tytgat et al. 2023). Antarctic lakes typically have very distinct microbial communities due to regional extinctions following the formation of the Antarctic ice sheets during successive glacial maxima combined with evolution in long-term isolation in scattered ice-free refugia (Pinseel et al. 2021, Tytgat et al. 2023). This distinctiveness is further reinforced by the geographic isolation of the continent by the Southern Ocean since about 25 million years (Clarke et al. 2005). By contrast, Arctic biota were able to migrate to ice-free regions south of the Northern Hemisphere ice sheets and recolonized the Arctic after glacial maxima, resulting in fewer regional extinctions and consequently more diverse food webs (Pointing et al. 2015).

The distinct evolutionary histories of polar microbiota and differences in food webs raise the question as to what extent microbial mat communities are also functionally differentiated between the Arctic and (sub-)Antarctic. However, polar shotgun metagenomics studies are rare and often restricted to one lake or one of the polar regions. As a result, the functional potential of microbial mat communities and their possible regional differentiation is poorly understood. One shotgun metagenomics analysis comparing both poles, namely microbial mats in ponds on Arctic and Antarctic ice shelves, revealed a high degree of similarity in functional genes, dominated by genes related to cellular and energy metabolism (Varin et al. 2012). However, some significant regional differences were observed. Antarctic samples were enriched in Photosystems I and II reads, alternative sigma factor stress response regulation genes and fatty acid desaturases, while in Arctic samples more trehalose biosynthesis genes and copper homeostasis genes were found. EPS biosynthesis genes were found in both regions and were mostly linked to Cyanobacteriota (Varin et al. 2012). The other available metagenomics data from polar lakes are those from deep sediments in Lake Hazen in the High Arctic (Ruuskanen et al. 2020), from different water depths in the Antarctic meromictic Ace Lake (Lauro et al. 2011), and from the water column and deep sediments in Lake Untersee (Koo et al. 2018, Wagner et al. 2022). These studies similarly showed the dominance of genes involved in cellular metabolism, nutrient cycling and genes coding for cold-induced proteins, indicating the adaptation of these communities to cold stress and nutrient starvation (Lauro et al. 2011, Koo et al. 2018, Ruuskanen et al. 2020, Wagner et al. 2022). In addition, genome-resolved metagenomics recovered 37 metagenome-assembled genomes of Cyanobacteriota from the same mats included in this study, further characterizing the cyanobacterial diversity in these mats (Pessi et al. 2023).

In this study, we compared the functional genetic potential of benthic microbial mats in the euphotic zone of Arctic, sub-Antarctic, and Antarctic lakes varying in environmental properties (Table S1) using shotgun metagenomic sequencing. We hypothesize that (i) genes crucial for adaptation to cold and oligotrophic polar conditions are prevalent in all samples, and (ii) known differences in environmental conditions and microbial communities between different polar lakes, including temperature, salinity, vegetation, taxonomic composition, and food web structure, are reflected in the functional genetic potential of these microbial communities.

## Methods

### Sample collection

**Figure 1:**
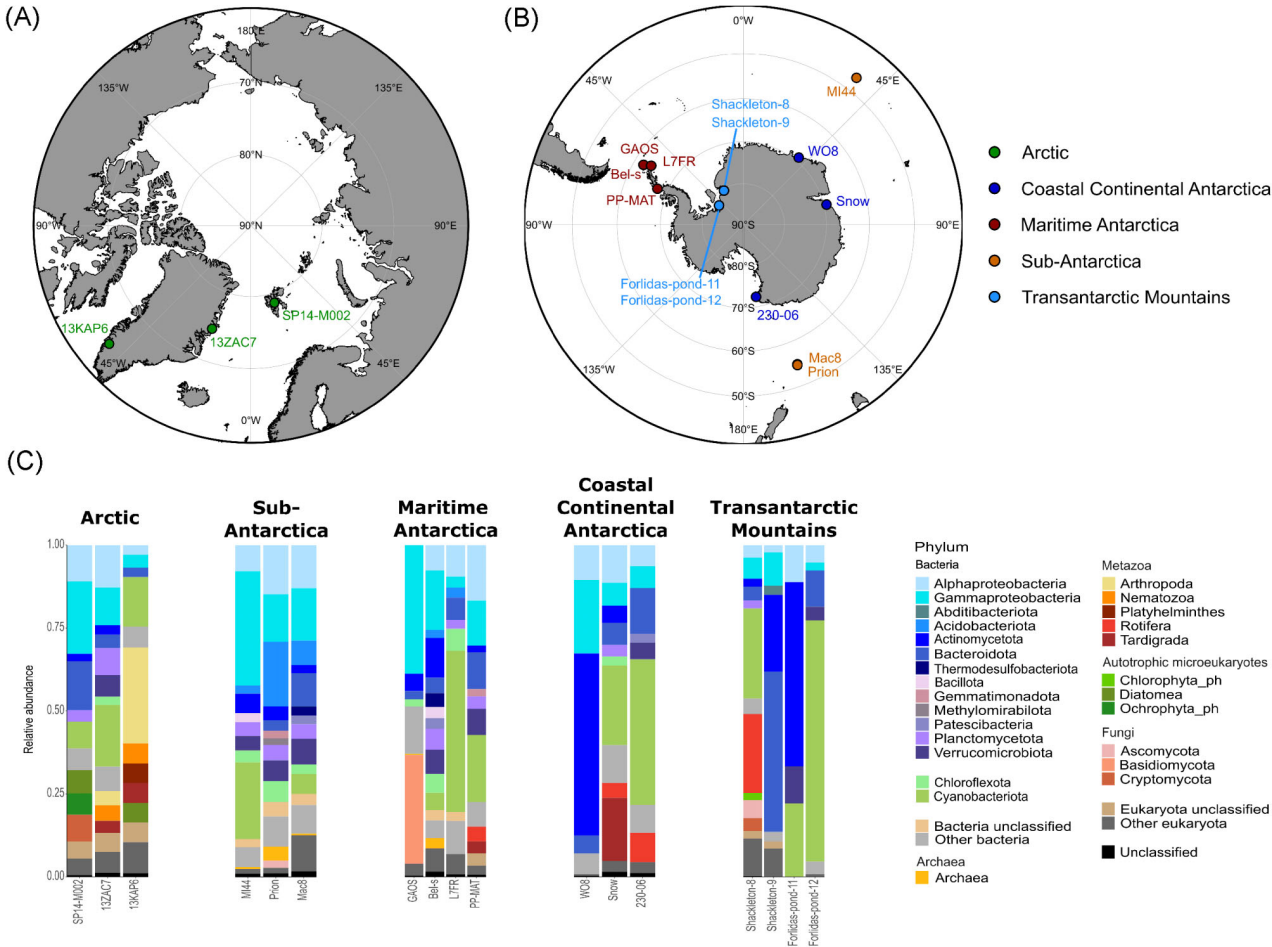
Map of the 15 studied lakes in (A) the Arctic and (B) sub-Antarctic and different regions of Antarctica. Sample information and environmental measurements can be found in Table S1. (C) Barplot of the community composition for the major phyla or classes with a relative abundance of at least 2% in the sampled microbial mats. Taxa are shown at the phylum level, except Pseudomonadota which are shown at the class level. Phyla with a relative abundance of <2% per sample are binned in ‘Other bacteria’ and ‘other eukaryota’. Patescibacteria is shown using the nomenclature adopted by the database and corresponds to the candidate phylum Candidatus Patescibacteria. Samples are grouped by geographic region.

In total, 17 microbial mat samples were analysed from the euphotic zone in 15 lakes from three main polar biogeographic zones, namely the Arctic, sub-Antarctica, and Antarctica, and varying in climatic and environmental conditions (Fig. 1, Table S1). A description of the study sites can be found in Hodgson et al. (2010), Pessi et al. (2023), and Watcham et al. (2011). Sampling was described in Tytgat et al. (2023). Briefly, benthic microbial mats from 15 different lakes were sampled in the littoral ice-free zone (± 20 cm from the lake shore) (12 lakes) using a sterilized spatula. Microbial mat samples from deeper parts (>1 m but never >6.5 m deep) of the euphotic zone, i.e. sunlit zone, in five lakes were collected using gravity corers. The upper one centimeter of the core was consecutively aseptically collected using a sterilized spatula and a high-precision extruder and slicing system designed to subsample sediment cores retrieved using a UWITEC gravity corer equipped with a ball core catcher. This approach ensures the preservation of sediment stratification by keeping core tubes upright during the slicing process. In each lake, one sample was taken, except for the lakes in the Transantarctic mountains in which a sample was taken from both the littoral zone and the deeper part (water depth >1 m). Samples were stored in the dark at −20°C until DNA extraction.

In the Arctic region, one lake was selected in South–West Greenland (sample name 13KAP6), one in North–East Greenland (sample name 13ZAC7) and one in Svalbard (sample name SP14-M002 Nordenskioldbreen) (Fig. 1A). From the sub-Antarctic islands, in Macquarie Island two lakes (sample name Mac8 and Prion Lake) and in Marion Island one lake (sample name MI44) were sampled. In Antarctica, nine lakes were studied, namely four from the Antarctic Peninsula (the Maritime Antarctic biogeographic region) [Gaoshan Lake (sample GAOS, King George Island), Belle Lake (sample Bel-s, King George Island), sample L7FR (Vega Island), and Pourquoi-pas Lake (sample PP-MAT, Pourquoi-pas Island)], and five from the Continental Antarctic biogeographic region, including three from coastal regions in East Antarctica [Snowbowl lake (sample Snow), West Ongul Island (sample WO8), and Lake North (sample 230–06)] and two from the Transantarctic Mountains, namely Lundström lake (Shackleton Range) and Forlidas Pond (Dufek Massif) (Fig. 1B). From the Transantarctic Mountains lakes, both a littoral sample (samples Shackleton-8 and Forlidas-pond-11) and a sample from a deeper part characterized by brackish or hypersaline waters (sample Shackleton-9, respectively, Forlidas-pond-12) were taken. The cyanobacterial diversity within Lundström Lake and Forlidas Pond was already studied in Fernandez-Carazo et al. (2011). A macroscopic description of each sample and information on the presence of vegetation in the catchment of the lakes is given in Table S1. To ensure comparability between biogeographic zones, a similar number of samples was selected per zone, yet this inevitably resulted in the inclusion of samples from lakes with different environmental properties in these various zones. For example, saline lakes were restricted to the Transantarctic mountains and lakes with vegetated catchments to Arctic and sub-Antarctic regions (Table S1)

Environmental data were collected as described in Tytgat et al. (2023). Specific conductance and pH were measured *in situ*. An overview of sample information and sample type (littoral or microbial mat), as well as measured environmental parameters, including pH and specific conductance, is given in Table S1. The lakes from Greenland, Svalbard, the sub-Antarctic, and to a lesser extent, the Maritime Antarctic have vegetated catchments, including microalgae, mosses, and angiosperms, while the lakes in the coastal Continental Antarctic regions and the Transantarctic Mountains have no active catchment vegetation.

### DNA extraction and metagenomics sequencing

DNA extraction is described in Tytgat et al. (2023). In brief, environmental DNA was removed according to Corinaldesi et al. (2005) and intracellular DNA was subsequently extracted according to Zwart et al. (1998). Briefly, cells were mechanically lysed by adding zirconium beads and DNA was extracted using a phenol–chloroform-based protocol with ethanol precipitation. Library preparation was performed using the Nextera XT kit following the manufacturer’s instructions (Illumina, USA). The fragmentation step was done at 55°C for 5 min. Nextera XT indices were used in the tagmentation (fragmentation and tagging) step. DNA quality and quantity were checked using a BioAnalyzer (Agilent). Samples were pooled equimolarly and sequenced on three HiSeq 2000 (Paired End 2 × 100 bp) runs at the Eurofins Genomics facilities (France).

### Sequence quality control and processing

Quality control of the sequencing reads was done with FastQC (Andrews 2010). Quality trimming of the raw sequencing reads was done with Trimmomatic (Bolger et al. 2014) using the following parameters: ILLUMINACLIP 2 : 30 : 10, LEADING:3, TRAILING:3, SLIDINGWINDOW:4 : 20, and MINLEN:50. Quality filtered reads were processed using the SqueezeMeta pipeline version 1.3.1 (Tamames and Puente-Sánchez 2019) in seqmerge mode using the following parameters: -a spades and -map bwa. Briefly, contigs were assembled separately for each sample with SPAdes (Bankevich et al. 2012). Individual assemblies were merged with minimus2 (Treangen et al. 2011). Ribosomal RNA (rRNA) and gene prediction were done with barrnap (Seemann 2014) and prodigal (Hyatt et al. 2010), respectively. Predicted open reading frames (ORFs) were functionally annotated against the KEGG version 58 (Kanehisa and Goto 2000) and eggNOG version 4.5 (Huerta-Cepas et al. 2016) databases using DIAMOND (Buchfink et al. 2015). Additionally, ORFs were annotated against the PFAM (Finn et al. 2014) database using HMMER (Eddy 2009). Taxonomic annotation of the ORFs was done against the NCBI nr database release 240 using DIAMOND (Buchfink et al. 2015). Predicted 16S and 18S rRNA sequences were taxonomically annotated using the RDP Bayesian classifier implemented in the mothur (version 1.48.0) classify.seqs command (Schloss et al. 2009) against the SILVA 138.1 database (Quast et al. 2013, Yilmaz et al. 2014). Throughout this manuscript, functional reads refer to predicted ORF sequences, while taxonomic composition is based on predicted 16S and 18S rRNA gene sequences.

### Statistical analysis

Data visualization and statistical analyses were done in the R statistical language system (R Core Team 2022) (version 3.6.3). The R package SQMTools version 1.6.0 (Puente-Sánchez et al. 2020) was used to load the data into R and for downstream analysis. To study the functional genetic potential, both KEGG pathways and COG functional categories were studied. In addition, 213 different marker genes (Table S2) involved in important processes, such as nutrient cycling and metabolism, stress response, remineralization of organic material and biofilm formation were filtered from the data using their corresponding KEGG ID. The marker genes were selected based on a literature search to identify the most commonly used marker genes for the selected processes. The raw abundances were normalized to copy numbers using the median abundance of 10 universal single-copy genes (Table S3), which are constitutively expressed across different conditions (Salazar et al. 2019, Puente-Sánchez et al. 2020). Copy numbers were calculated by dividing the transcript per million (TPM) values of each gene by the median TPM of 10 universal single-copy genes (Table S3), as suggested by the SQMTools package developers (Puente-Sánchez et al. 2020). TPM values were calculated as part of the SqueezeMeta pipeline and are defined as the number of times a feature (e.g. contig or gene) is found when randomly sampling 1 million features, given the abundances of the different features in a sample (Puente-Sánchez et al. 2020). Copy number normalized KEGG ID and COG ID abundances were summed to obtain a total abundance for every pathway. The R package pheatmap version 1.0.12 (Kolde 2022) was used to generate the heatmaps of pathways and marker genes. Copy number normalized abundances were Z-transformed for visualization using the scale parameter from the *pheatmap* function.

To investigate the presence of some important pathways in the different regions, boxplots were made per region and pathway. Raw abundances of the selected marker genes (Table S2) and COG IDs were summed to obtain a total abundance for every selected pathway and COG functional category. Total abundances were transformed to relative abundances.

Following Sunagawa et al. (2015) we defined a set of core functions. Raw COG ID abundances were rarefied to the smallest library size. A COG ID was considered to be part of the set of core functions if at least one read from each sample was mapped to a gene annotated to that COG ID.

Given the low sequencing depth of the sample Forlidas-11 (22 660 reads), this sample was removed for plotting the boxplots and defining the set of core functions. The extremely low read number in this sample likely resulted from a technical issue.

Kruskal–Wallis rank sum tests were done with the *kruskal.test* function from the stats package version 3.6.3 (R Core Team 2022) to identify differences in the abundance of pathways and functional categories across regions. Multiple pairwise comparisons were performed with the *dunn_test* function from the rstatix package version 0.7.2 (Kassambara 2025) to calculate pairwise comparisons between groups using Benjamini–Hochberg corrections for multiple testing.

Community composition was studied based on the predicted 16S and 18S rRNA sequences. Relative abundances of each taxon were calculated. Barplots were made using the R package ggplot2 version 3.5.0 (Wickham et al. 2022).

## Results

Across all samples, a total of 857 234 388 reads (mean per sample 50 425 552 ± 41 794 556 SD) were retained after quality trimming. The average read number per sample was within the range typically found in similar metagenomics studies from microbial mats or sediments (Gutiérrez-Preciado et al. 2018, Kindler et al. 2020, Centurion et al. 2021). The final assembly consisted of a total of 5 159 038 ORFs of which 99.7% (5142 395 ORFs) were predicted as coding sequences. The remainder were predicted as rRNA, transfer RNA (tRNA), or transfer–messenger RNA (tmRNA). The assembly comprised 4420 044 contigs, with N50 and N90 values of 479 and 225, respectively.

From the predicted coding sequences, a total of 40% (2077 218 ORFs) could be assigned a KEGG ID and 59% (3012 444 ORFs) could be assigned a COG ID. On average, 55 ± 21% (SD) of the quality filtered reads could be mapped back to the final assembly (Fig. S1), which implies that still a considerable part of the functional gene diversity is not represented in the final assembly.

### Taxonomic analysis

A total of 0.06% of all ORFs in the final assembly were predicted as rRNA sequences (2985 ORFs). The microbial mat communities were dominated by bacteria [average relative abundance 81 ± 19% (SD)] and Eukaryota [average relative abundance 18 ± 19% (SD)]. Archaea accounted only for a small proportion of the rRNA data [average relative abundance 0.5 ± 1.2% (SD)]. A detailed overview of the taxonomic composition of the mats, based on amplicon sequencing of 16S and 18S rRNA genes, is described in Tytgat et al. (2023) and a detailed description of the cyanobacterial diversity is described in Pessi et al. (2023).

The bacterial fraction of the microbial mat communities was dominated by Pseudomonadota and Cyanobacteriota, while the eukaryotic fraction mostly consisted of Rotifera and Tardigrada. Pseudomonadota were the most abundant phylum in the microbial mats from the Arctic, sub-Antarctica, and North–West Antarctic Peninsula. In the microbial mats from Coastal Continental Antarctica, the Transantarctic Mountains and North–East Antarctic Peninsula, Cyanobacteriota were generally more abundant (Fig. 1C). Actinomycetota were highly abundant in WO8, Shackleton-9 and Forlidas-pond-11 (Fig. 1C). Bacteroidota were more abundant in the deeper, more saline samples from Lundström Lake and Forlidas Pond (Shackleton-9 and Forlidas-pond-12) compared to the top littoral samples (Shackleton-8 and Forlidas-pond-11). Arthropoda were only found at high abundance in the Arctic mat 13KAP6 and to a lesser extent in 13ZAC7. While in 13KAP6 these were mainly classified to the order Diptera, the Arthropoda found in 13ZAC7 were mostly classified to the order Harpacticoida or Maxillopoda unclassified at the order level. Basidiomycota were present in high abundance in GAOS only (Fig. 1C). These sequences were related to the family Mrakiaceae, a family of yeast-like fungi. An overview of the most abundant classes found in each sample is shown in Fig. S2.

### Functional potential of microbial mats

**Figure 2:**
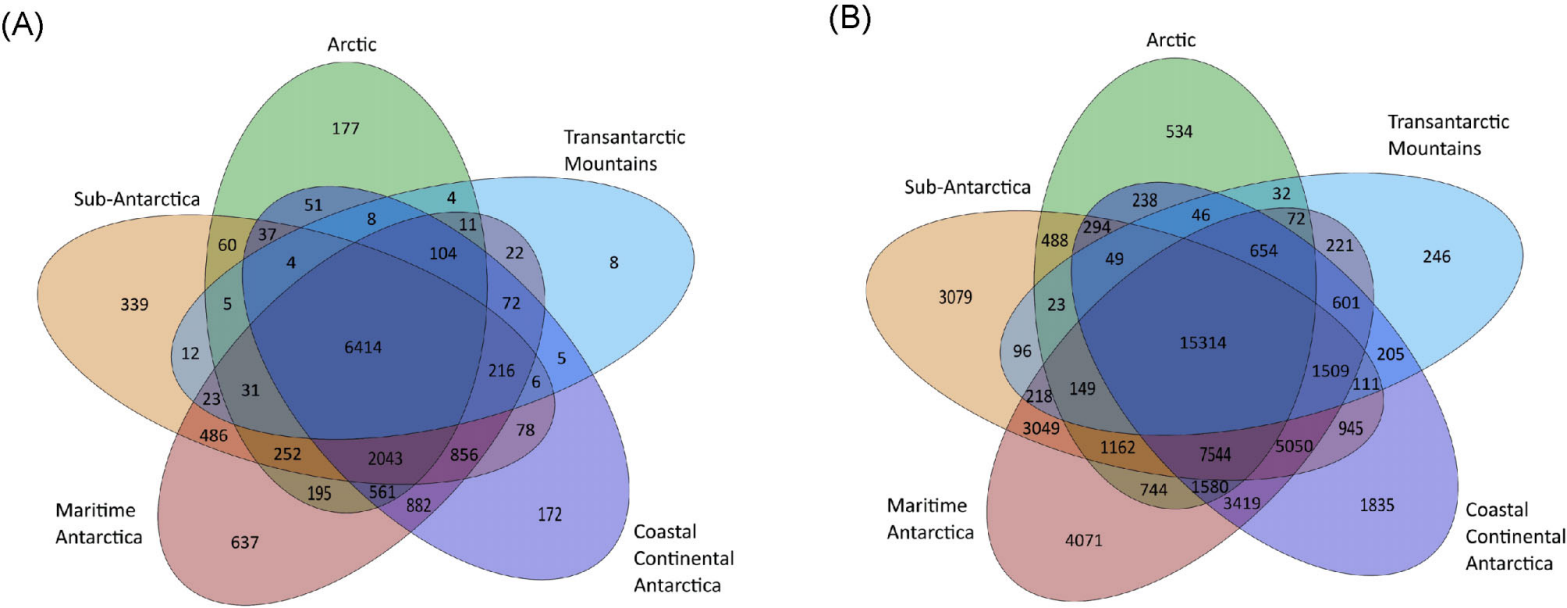
Shared and unique KEGG (A) and COG (B) IDs between the different regions.

A large number of KEGG (46.6%) and COG (29%) IDs were shared between all the different regions, suggesting a high proportion of shared functions between the microbial mats. However, the sub- and Maritime Antarctic mats had a higher number of unique KEGG and COG IDs compared to the other regions (Fig. 2), and these were mainly involved in signal transduction and transport functions, and DNA replication and transcription functions (Tables S4 and S5).

**Figure 3:**
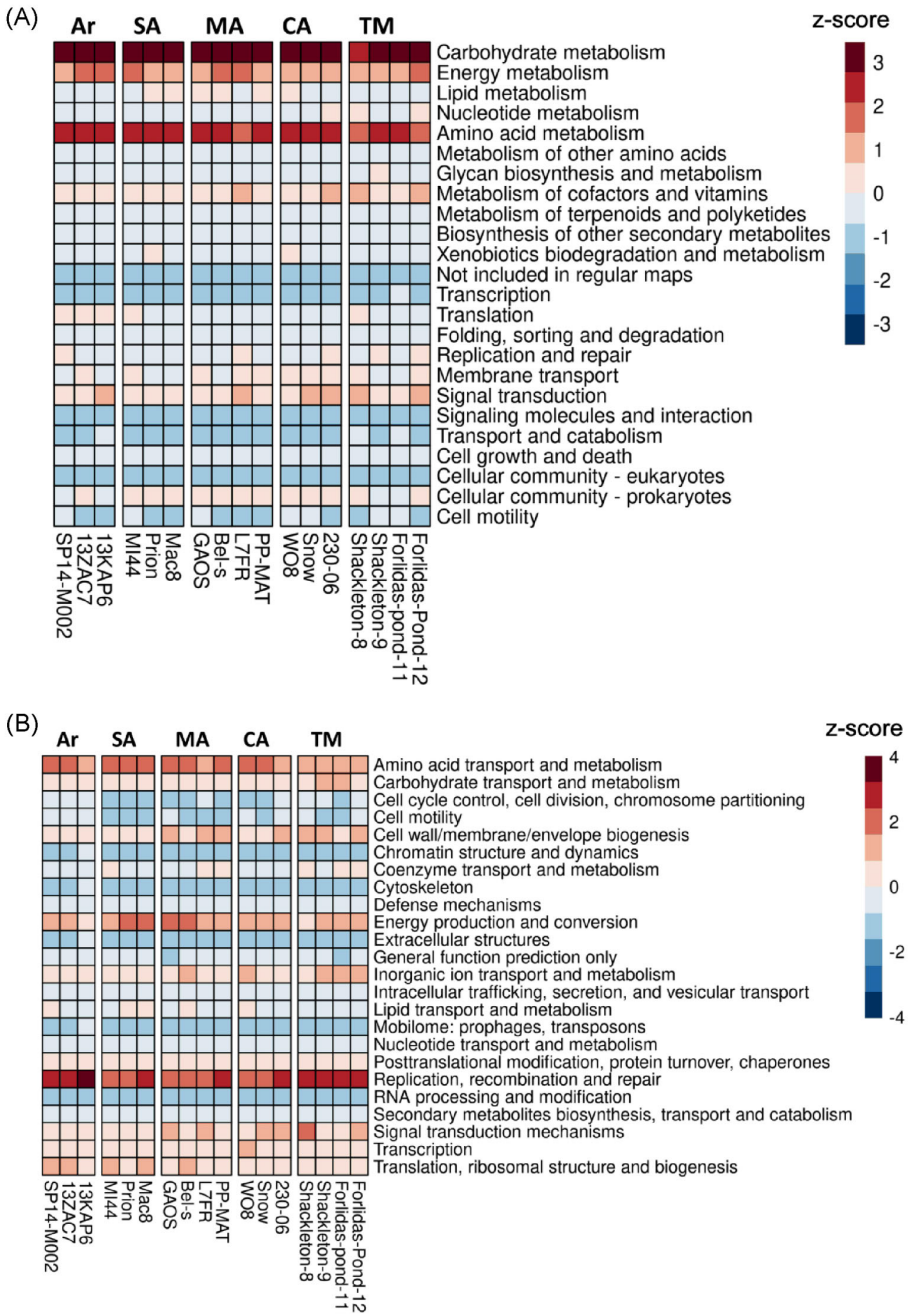
Heatmap of abundances of KEGG pathways (A) and COG functional categories (B). Abundances were normalized to copy numbers and z-transformed by column. Red colours represent high normalized abundances, blue colours represent low normalized abundances. Normalized gene abundances were summed to obtain a total abundance for each KEGG pathway or COG functional category. The COG functional category ‘Function unknown’ was removed in (B). A plot showing all categories is depicted in Fig. S3. Samples are grouped by geographic region. Ar = Arctic, SA = sub-Antarctica, MA = Maritime Antarctica, CA = Coastal Continental Antarctica, and TM = Transantarctic mountains.

A high degree of similarity was also observed between the regions at the level of KEGG pathways (Fig. 3A) and COG functional categories (Fig. 3B, Fig. S3). Carbohydrate, amino acid, and energy metabolism pathways are the most abundant ones across all mats, followed by genes involved in signal transduction. Genes involved in membrane transport and biogenesis, and replication and repair also showed high abundances in most mats. When looking at COG functional categories, up to a mean relative abundance per sample of 27.8% of the reads were annotated to COG IDs with an unknown function (Fig. S3).

**Figure 4:**
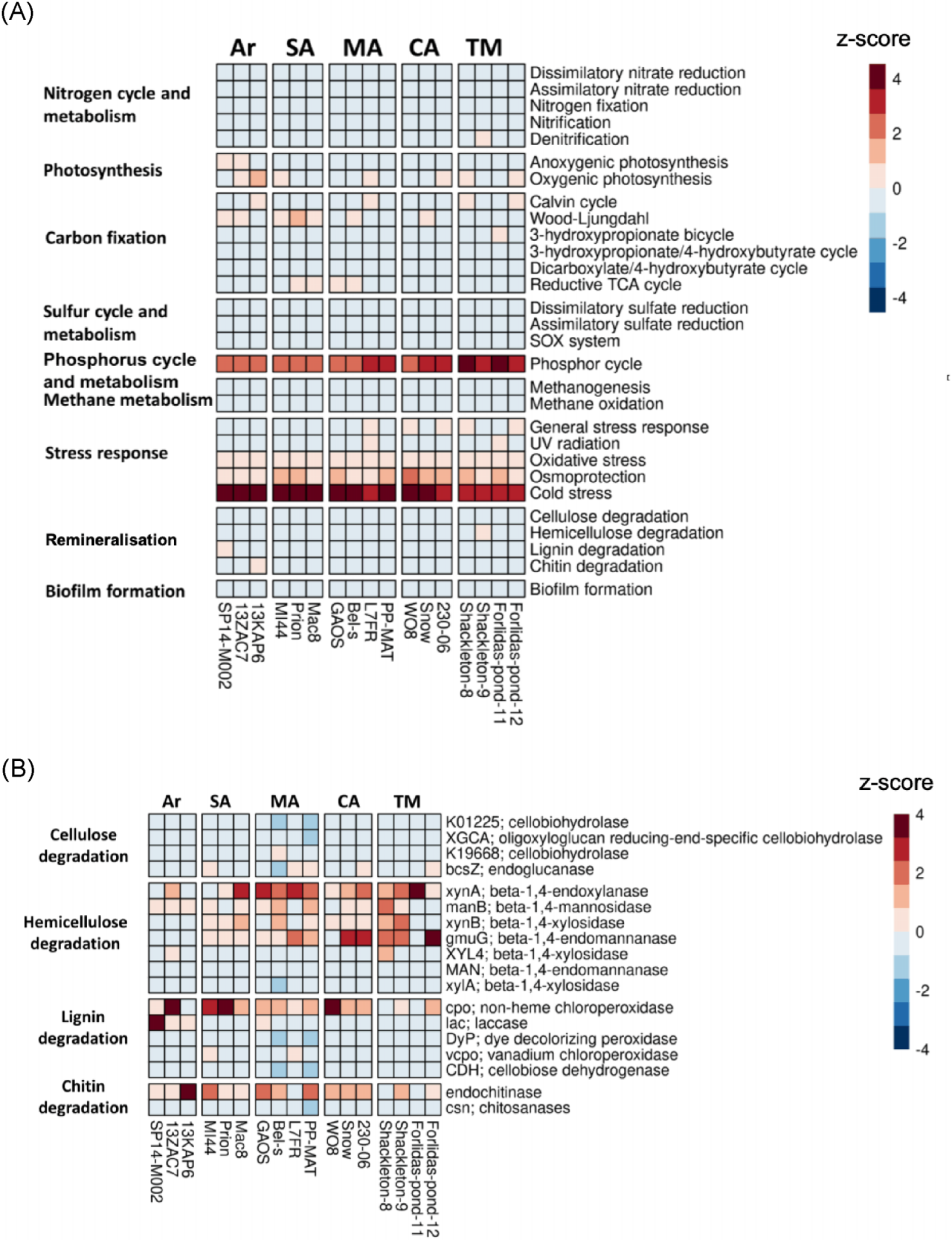
(A) Heatmap of abundances of selected functional processes involved in nutrient cycling and metabolism, stress response, remineralization of organic material, and biofilm formation. Abundances were normalized to copy numbers and z-transformed by column. Red colours represent high normalized abundances, blue colours represent low normalized abundances. Normalized gene abundances were summed to total abundance for each process. Samples are grouped by geographic region. Ar = Arctic, SA = sub-Antarctica, MA = Maritime Antarctica, CA = Coastal Continental Antarctica, and TM = Transantarctic mountains. (B) Heatmap of genes involved in remineralization of organic material. Abundances were normalized to copy numbers and z-transformed by column. Normalized gene abundances were summed to total abundance for each process. Samples are grouped by geographic region. Ar = Arctic, SA = sub-Antarctica, MA = Maritime Antarctica, CA = Coastal Continental Antarctica, and TM = Transantarctic mountains.

Specific pathways involved in stress response and in important ecosystem functions, including nutrient cycling, remineralization of organic material, and biofilm formation, were further investigated in more detail by filtering out a selection of 213 marker genes for those pathways based on a literature search (Fig. 4A). The studied pathways were in general evenly abundant in all mats, yet some differences between samples were present. Stress response genes, and especially cold stress response genes, showed the highest relative abundance in all sampled lakes. The majority of the studied (cold) stress response marker genes were present in all mats (Fig. S4). The sequences related to cold stress response genes were annotated to a diversity of taxonomic groups, although most genes had a bacterial annotation (Table S7) with a particularly high contribution of Pseudomonadota and Bacteroidota across the different regions. Cyanobacteriota were also found as an important source of these genes, with the exception of the sub-Antarctic mats in which Acidobacteriota were their most important producers. However, some caution needs to be taken when considering the taxonomic assignment of functional genes given the lack of well characterized reference genomes or gene sequences for various nonmodel species (Shakya et al. 2019). The selected (cold) stress response marker genes included genes encoding DNA replication initiator (DnaA), molecular chaperones (DnaK and DnaJ), recombination factor A (RecA) and DNA gyrase (GyrA). Translation initiation factors (InfA, InfB, and InfC), involved in protein biosynthesis, were also present. Pyruvate dehydrogenases (AceE and AceF), also found to be induced upon cold and other stresses (Koo et al. 2018), were observed in most mats. In contrast, among cold shock proteins, only the gene encoding RNA chaperone (CspA) was found, and this was mainly in sub- and Maritime Antarctica and the sample WO8, and to a lesser extent in the saline microbial mat sample from Shackleton Range (Shackleton-9) (Fig. S4). Sequences linked to this gene were mainly annotated to Pseudomonadota and Actinomycetota. DNA binding protein subunit HU-alpha (hupA), the most abundant nucleoid-associated protein in many bacterial cells and involved in DNA stabilization (Strzałka et al. 2024), was only found in samples from sub- and Maritime Antarctica while the subunit HU-beta (hupB) was found in mats from all regions (Table S6). The RNA binding protein Cold-shock-domain protein A (CSDA), involved in gene regulation after cold shock (Xu et al. 2017), was present only in Arctic and sub-Antarctic mats, while the cold-shock induced palmitoleoyltransferase (lpxP), that increases the outer membrane fluidity (Vorachek-Warren et al. 2002), was only found in sub-Antarctica. The majority of these (cold) stress response sequences were mainly annotated to Pseudomonadota, however, CSDA sequences were predominantly associated with unclassified Eukaryota.

Genes involved in phosphorus cycling and metabolism were also abundant in all mats, especially genes encoding high-affinity phosphate transporter Pst system were prevalent in most mats (Fig. 4A, Fig. S5). Furthermore, polyphosphate kinase (ppk) and phosphate regulon (PhoR, PhoB, and PhoP) genes were also abundant in most samples. Sequences from phosphorus cycling and metabolism genes were mainly annotated to Pseudomonadota, Cyanobacteriota, and Bacteroidota, except in sub-Antarctica where Acidobacteriota were more important contributors (Table S8).

Genes involved in nitrogen cycling and metabolism were very rare in all mats, despite the high abundance of Cyanobacteriota in many samples (Fig. 4A, Fig. S6A). From the nitrogen cycling marker genes, those involved in assimilatory nitrate reduction and denitrification appeared most abundant (Fig. S6A). Denitrification genes had a higher occurrence in the Antarctic samples compared to the Arctic ones, however, this difference was not statistically significant (Wilcoxon test, *P*-value = .139). Nitrogen fixation genes were generally less abundant, with the highest abundances found in the Arctic lakes. However, regional differences in nitrogen fixation gene abundance were also not significant (Wilcoxon test, *P*-value = .075).

The most important carbon fixation pathways were the Calvin cycle, Wood–Ljungdahl pathway, and the Reductive tricarboxylic acid (rTCA) cycle (Fig. 4A, Fig. S6B). The Calvin cycle is the most well-known mechanism of biological carbon fixation and is the only carbon fixation pathway occurring in eukaryotes (Berg 2011, Hügler and Sievert 2011). The Wood–Ljungdahl pathway is present in both bacteria and archaea and is considered one of the most ancient carbon fixation pathways (Hügler and Sievert 2011, Adam et al. 2018). The rTCA cycle or reverse Krebs cycle is widespread among anaerobic or microaerobic bacteria (Hügler and Sievert 2011). In the Arctic and Continental Antarctic mats, the Calvin cycle (Wilcoxon test, *P*-value = .938) and Wood–Ljungdahl pathway (Wilcoxon test, *P*-value = .015) were the most important carbon fixation pathways, while in the sub- and Maritime Antarctic mats the Wood–Ljungdahl pathway and rTCA cycle (Wilcoxon test, *P*-value = .265) were more important (Fig. S6B). Calvin cycle genes were annotated mostly to Cyanobacteriota and Pseudomonadota, and eukaryotic sequences were mainly annotated to Bacillariophyta. Gene sequences involved in the Wood–Ljungdahl pathway were mainly linked to Euryarchaeota, Pseudomonadota, and Gemmatimonadota, whereas Pseudomonadota and Nitrospirota contributed more to genes involved in the rTCA cycle.

### Core and noncore functions across polar regions

**Figure 5:**
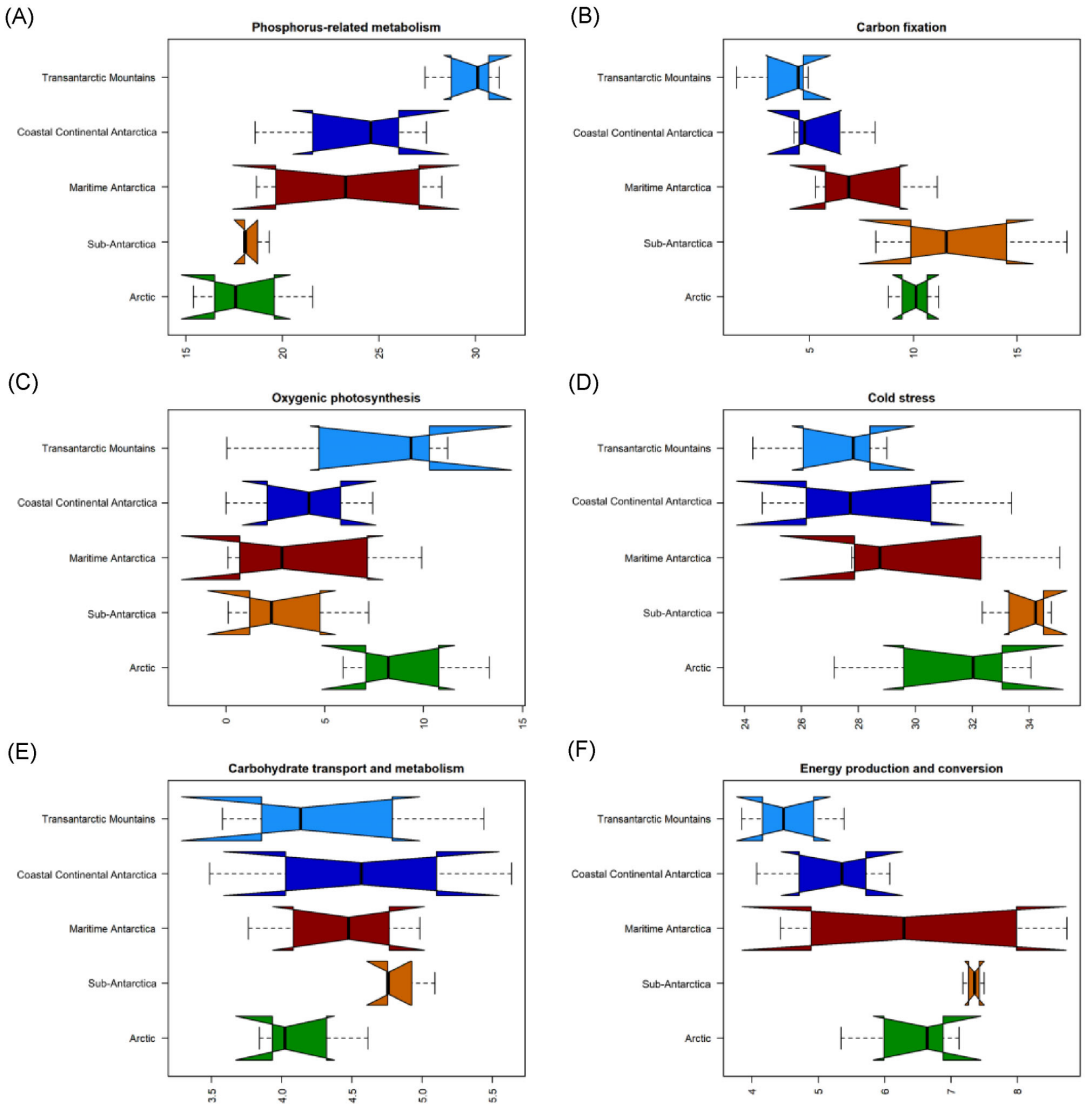
Boxplots showing the distribution of the relative abundances of selected marker genes and COG functional categories involved in (A) phosphorus cycling and metabolism, (B) carbon fixation, (C) oxygenic photosynthesis, (D) cold stress response, (E) carbohydrate transport and metabolism, and (F) energy production and conversion per region. Whiskers represent the minimum and maximum value within a range of 1.5 times the interquartile range. No overlap between the notches of boxplots indicates possible significant differences between the median values of the respective groups.

The fraction of core functions, defined here as COG IDs found in all samples (cf. Sunagawa et al. 2015), consisted of 57 ± 9% (SD) of the functional reads. After removing this fraction and the unclassified fraction [24 ± 12% (SD) of the functional reads], 19 ± 5% (SD) remained as the noncore fraction. Functional categories within the core fraction were evenly distributed across the different regions (Fig. S7A). A majority of the genes within the noncore fraction encode for genes with an unknown function (Fig. S7B). Within the rest of the noncore fraction, genes involved in signal transduction were generally found to be more abundant in the Antarctic mats compared to the Arctic mats’ samples (Fig. S7B) but this difference was not statistically significant (Wilcoxon test, *P*-value = .508). This functional category includes, among other things, protein phosphatases and kinases and regulator proteins, including some of the abovementioned phosphorus cycling and metabolism marker genes. There was also a trend towards increasing relative abundance of phosphorus cycling marker genes with increasing latitude in the Southern Hemisphere (Wilcoxon test, *P*-value = .050), and their abundance in the Arctic lakes is more or less similar to that in the sub-Antarctic (Fig. 5A). This was also in agreement with the heatmap of all the studied pathways using marker genes (Fig. 4A).

The functional categories carbohydrate transport and metabolism (Wilcoxon test, *P*-value = .60), and energy production and conversion (Wilcoxon test, *P*-value = .127) showed a decreasing trend in relative abundance with increasing latitude in the Southern Hemisphere (Fig. 5E and F). The abundance of these categories in the Arctic mats was again similar to that in the sub-Antarctic. The same trend was found for the carbon fixation marker genes (Fig. 5B), which are included in the carbohydrate transport and metabolism functional category, and this trend was statistically significant (Wilcoxon test, *P*-value = .025).

Genes involved in oxygenic photosynthesis were more abundant in the Arctic and Coastal Continental Antarctic mats’ samples (Figs 4A and 5C) but this difference was not statistically significant (Wilcoxon test, *P*-value = .480). Cold stress response marker genes had a somewhat lower relative abundance in the Antarctic samples compared with the Arctic and sub-Antarctic mats, but this difference was also not statistically significant (Wilcoxon test, *P*- value = .227) (Figs 4A and 5D).

### Links between functional genetic potential and local conditions

Some of the differences in the functional potential between the different mats’ samples could be related to local conditions in the different lake ecosystems. Notably, genes involved in the remineralization of organic material were relatively more abundant in the lakes with vegetated catchments, such as those in sub- and Maritime Antarctica (Fig. 4). The prevalence of these genes could be linked to the type of vegetation in the catchment as those involved in hemicellulose (typically associated with plant cell walls) degradation were relatively more abundant in the Maritime Antarctic and Transantarctic Mountain mats, whereas genes linked to lignin (typically associated with plant cell walls and vascular tissue) and chitin (typically associated with insects, crustaceans and fungi) degradation were relatively more important in the Arctic mats, particularly the samples SP14-M002 and 13KAP6 (Fig. 4B). However, the differences between regions were not statistically significant for hemicellulose (Wilcoxon test, *P*-value = .642) and chitin (Wilcoxon test, *P*-value = .701) degradation genes. The abundance of lignin degradation genes differed significantly between regions (Wilcoxon test, *P*-value = .0049), with lower abundances observed in Transantarctic Mountains samples compared to the other regions. Sequences linked to cellulose and hemicellulose degradation were annotated to a diversity of phyla, such as Acidobacteriota, Bacteroidota, Chloroflexota, Cyanobacteriota, Planctomycetota, and Pseudomonadota. Lignin degrading genes were primarily annotated to Pseudomonadota, Bacteroidota, and Actinomycetota, while chitin degradation genes were mostly linked to Bacteroidota and Pseudomonadota in the Arctic mats. However, in 13KAP6 these sequences were mainly coming from Arthropoda and Gammaproteobacteria. In the sub- and Maritime Antarctic mats Bacteroidota, Pseudomonadota, and Actinomycetota were the main contributors, whereas in the microbial mat samples from the Transantarctic Mountains these sequences were mostly annotated to Bacteroidota.

## Discussion

Our analyses revealed that a considerable part of the functional reads (57%) were shared between the 17 studied microbial mats and can thus be regarded as core functions of the studied communities. Genes involved in carbohydrate, amino acid, and energy metabolism were very abundant in all mats (Fig. 3, Fig. S3), which is consistent with previous metagenomic studies from diverse environments, including Arctic meltwater ponds (Varin et al. 2010) and tropical coral reef mats (Cissell and McCoy 2021). The dominance of these housekeeping genes can be explained by their key roles in processes crucial for cell growth and maintenance, including cellular energy production, and production and transport of amino acids and sugars (Kanehisa and Goto 2000). Genes involved in both aerobic and anaerobic metabolic pathways were also present in all samples, which likely reflects the presence of specific microbial consortia adapted to thrive in the different microzones of the mats, which have contrasting environmental conditions, or to seasonal variations in abiotic properties. Indeed, microbial mats are highly structured and consist of different microzones with biochemical and physical gradients. During winter, photosynthesis is low or absent in these mats, while remineralization of organic matter and other metabolism pathways, such as anaerobic pathways, become more important (Varin et al. 2010).

Stress response genes were also ubiquitous and attained a mean relative abundance of >30% across all samples (Fig. 4A, Fig. S4). While the studied stress response genes are generally involved in responses to different types of stresses, the selected cold stress response genes have been used as marker genes for cold stress by other authors and are abundant in polar aquatic ecosystems (Varin et al. 2012, Lay et al. 2013, Koo et al. 2018). DNA gyrase (gyrA), DNA replication initiator (dnaA), and molecular chaperones (dnaK and dnaJ) were amongst the most abundant (cold) stress response genes in all studied mat samples and are known to be involved in cell survival at cold temperatures by stabilizing and ensuring the correct folding of DNA (Rodrigues and Tiedje 2008). Moreover, pyruvate dehydrogenase genes (aceE and aceF), involved in maintaining membrane fluidity at cold temperatures (Rodrigues and Tiedje 2008), were also abundant. Notably, only one cold shock protein (protein A, cspA) was present, primarily in the sub- and Maritime Antarctic mats. Cold shock proteins are important for translation at low temperatures and while they have been found in a wide diversity in Antarctic soils, and water and sediment samples from Lake Untersee, a similarly low diversity has been observed in cyanobacterial mats from Arctic and Antarctic ice shelf ponds (Varin et al. 2012). We therefore speculate that these proteins may be less important in the cold stress response of microbial mat communities from shallow lakes and ponds compared to soil, planktonic, and deeper sediment communities. Instead of relying on a single mechanism, such as cold shock response, these communities likely express a diverse set of genes to cope with the broad range of stresses in the highly dynamic habitats in ponds and shallow lakes. The rarity of cold shock proteins can, however, also be related to the low mapping percentage of the gene assemblies (Fig. S1), indicating that not all the functional diversity is captured in the assembly. Pseudomonadota and Cyanobacteriota contributed considerably to the (cold) stress response gene sequences, likely reflecting their high abundance in the studied mats (Fig. 1C), as also observed by Tytgat et al. (2023). However, in sub-Antarctica, Acidobacteriota contributed more to the cold stress response genes, consistent with their higher abundance in the mats from this region (Fig. 1C, Table S7). This may possibly relate to their ability to degrade a broad set of oligo- and polysaccharides, and the influx of different organic molecules produced by the catchment vegetation (Kielak et al. 2016).

In addition to stress response genes, also genes involved in phosphorus cycling and metabolism were highly abundant in all samples (Fig. 4A, Fig. S5). Previous studies have shown the ability of microbial mat biota to recycle and scavenge nutrients as reflected in higher nutrient concentrations within mats compared to the overlying water (Vincent et al. 1993, Varin et al. 2010). Indeed, we observed a relatively high amount of alkaline phosphatases (phoA and phoD), ppk, phosphate regulon genes (phoB–phoR), and genes for the transport of different forms of phosphorus (Fig. S5). Alkaline phosphatases are typically induced by inorganic phosphorus starvation in bacteria. Polyphosphate kinase is involved in the accumulation of polyphosphate as an inorganic phosphorus reservoir (Santos-Beneit 2015). The high-affinity phosphorus transporter pst (pstSCAB) is an important factor in the sensing of phosphorus limitation, and the pstS gene is commonly used as a biomarker for monitoring phosphorus limitation (Santos-Beneit 2015, Pereira et al. 2019). Although some of these genes are also involved in intracellular processes, such as membrane integrity and intracellular signalling (Santos-Beneit 2015), the high relative abundance of these genes may reflect the oligotrophic nature of the lakes (Hodgson et al. 2010, Watcham et al. 2011, Pessi et al. 2023, Tytgat et al. 2023). More in particular, most of these genes are controlled by a two-component regulatory system, which controls cellular responses to low extracellular phosphate concentration and phosphorus assimilation (Varin et al. 2010, Santos-Beneit 2015). Combined, the high abundance of these genes could possibly reflect the potential of these microbial communities to scavenge phosphorus from the water column and recycle it within the mats as also observed in both marine (Varin et al. 2010, Campbell et al. 2020) and freshwater microbial mats (Varin et al. 2010).

Consistent with this, genes involved in signal transduction pathways, important in the microbial response to diverse environmental stresses, were also relatively abundant (Fig. 3B, Fig. S3). This could again reflect the importance of nutrient recycling by the mat communities (Varin et al. 2010, Dang and Lovell 2016). However, in contrast to genes involved in phosphorus cycling, nitrogen cycling genes were relatively less abundant (Fig. 4A). Assimilatory nitrate reduction and denitrification were probably the most important processes in Antarctic mats based on the relative abundance of genes involved in nitrogen cycling (Fig. S6A). Nitrogen-fixation genes showed strikingly low relative abundances, despite the presence of potential nitrogen-fixing species (e.g. those belonging to the family Nostocacea) in some mats, and were relatively more abundant in Arctic and sub-Antarctic mats. The oligotrophic nature of most polar lakes, and especially a high N:P ratio (Ellis-Evans 1996), can explain the relatively low amount of nitrogen-fixation genes and other nitrogen-cycling genes in these microbial mat samples compared to other functions. Additionally, because nitrogen fixation is temperature-dependent and energetically costly, the consistently low temperatures in polar regions might suppress this process (Ellis-Evans 1996, Varin et al. 2010).

### The functional potential of microbial mat communities may reflect local conditions

The studied lakes vary in environmental and climatic conditions, even within regions. These differences, including temperature, catchment vegetation, and consequently taxonomic composition and food web structure, likely shape the functional genetic potential of the microbial communities. By contrast, differences in salinity were not clearly reflected in the functional genetic potential of the studied microbial mats (Fig. 4A). It is possible that differences in salinity are reflected in the microbial activity while this study only analyses the presence of genes. Alternatively, this observation could also be related to the low mapping percentage of the assemblies.

In contrast to salinity, variation in climatic conditions and vegetation catchment could possibly be more closely linked to differences in functional genetic potential. For example, Continental Antarctica, including the Transantarctic Mountains, experiences harsher conditions than the other studied regions. As a result, one of the lakes (Lündstrom Lake) lacks vegetation in its catchments. In Forlidas Pond, dried cyanobacterial mats are present, yet not active as they were fossilized c. 4300–2250 year BP ago (Hodgson and Bentley 2012), and hence do not provide organic matter to the system. By contrast, the lakes in the other regions have vegetated catchments in which algae, mosses, and angiosperms thrive. These differences are likely reflected in the abundance of genes involved in the remineralization of organic matter. More in particular, lignin degrading genes, typically associated with vascular plants and mainly angiosperms (Ribeiro et al. 2023), were most important in systems with vegetated catchments in which higher plants occur. Surprisingly, they were also present in sample WO8, which has a barren catchment (Fig. 4B). These sequences were mainly annotated to Pseudomonadota and Acidobacteriota, which are also found abundantly in this sample (Fig. 1C). As mentioned above, our study analyses gene abundances rather than gene expression levels, implying that the presence of these genes in this Continental Antarctic lake might represent a genetic potential which is no longer expressed, but may become advantageous if environmental conditions change or if organisms disperse to other environments.

When lignin degrading genes were present, the catchment vegetation surrounding the Maritime and sub-Antarctic probably provides an additional allochthonous source of organic matter, which likely results in the selection for taxa being capable of C-remineralization. By contrast, hemicellulose-degrading genes, primarily associated with plant cell walls, including those of mosses (Roig-Oliver et al. 2021), were relatively more important in systems lacking vascular plant vegetation in their catchment (Fig. 4B). Chitin degrading genes were relatively abundant in most mats, which is probably related to chitin being the second most abundant biopolymer, which forms the structural component of the exoskeleton in arthropods and tardigrades and the cell walls of fungi (Gooday 1990). However, the producers of chitin degradation genes seemed to be different between samples. In the sub- and Maritime Antarctic, they were mainly linked to Bacteroidota, Pseudomonadota, and Actinomycetota, whereas in the deeper, more saline samples of the Transantarctic Mountains lakes, Bacteroidota were the main contributors, which is in line with their higher abundance in these samples (Fig. 1C). In the Greenland mats’ samples, these sequences were mainly linked to Arthropoda. In the mat from 13KAP6, most of these sequences were annotated to the order Diptera, while in the 13ZAC7 mat the order Harpacticoida (subclass Copepoda) and unclassified Maxillopoda were more abundant. This is congruent with the rRNA gene analysis, which revealed that Arthropoda were mainly abundant in the Greenland mats, but can also be related to the stochastic occurrence of particular macroscopic organisms in the samples, as the latter were too small to reflect the entire lake communities.

Catchment vegetation also influences lake nutrient levels, by providing an additional source of organic matter and additional nutrients via root exudates and photoautotrophs in surrounding wetlands (Jacoby et al. 2017, Camacho et al. 2022). Organic matter availability can, in turn, affect microbial carbon fixation. For example, Grüterich et al (2024) found that the genes involved in the reductive tricarboxylic acid (rTCA) carbon-fixation pathway were more expressed in wetlands high in organic matter. Given the vegetated catchments of sub- and Maritime Antarctic lakes, it can be hypothesized that these systems also have a higher allochthonous organic matter content, potentially explaining the relative importance of the rTCA pathway in these mats. Genes related to the rTCA cycle were mainly annotated to Alphaproteobacteria and Deltaproteobacteria, taxa known to be linked to this pathway (Hügler and Sievert 2011) and found abundantly in sub- and Maritime Antarctic mats (Fig. 1C). Furthermore, vegetated catchments in these regions may contribute to the predominance of *Mrakia* species (phylum Basidiomycota) in these samples, particularly in the sample GAOS. These cold-adapted fungi have already been isolated from a variety of cold environments, including the Arctic, Antarctica, and the European Alps (Xin and Zhou 2007, Thomas-Hall et al. 2010, Singh and Singh 2012, Tsuji et al. 2013, 2018) and are a major part of the fungal community of East Antarctic lake sediments (Tsuji et al. 2013). Given the ability of these species to degrade organic macromolecules, we hypothesize that they may benefit from the increased availability of diverse organic compounds in these lakes (Turchetti et al. 2008, Thomas-Hall et al. 2010). Nutrient availability within the mat environment can also impact microbial community functional potential. While genes involved in phosphorus cycling were present in all mat samples, the high-affinity transporter gene pstS and response regulator gene phoP were more abundant in the Continental Antarctic lakes, in particular in the samples from the Transantarctic Mountains (Fig. S5). This likely reflects the ultra-oligotrophic conditions of these glacial lakes (Laybourn-Parry and Pearce 2007).

Differences in environmental conditions are likely also reflected in microbial community composition and functional genetic potential. The prevalence in the studied microbial mats of genes involved in the Wood–Ljungdahl carbon fixation pathway (Fig. S6B), which is exclusively functional in anaerobic organisms (Hügler and Sievert 2011), might be related to anaerobic conditions in the deeper microbial mats or within particular zones inside the mats where oxygen from the surface does not penetrate, or anoxic conditions during extended periods of ice cover during winter (Ellis-Evans 1996, Varin et al. 2010). In our dataset, these genes were annotated, amongst others, to Euryarchaeota, Pseudomonadota, Bacillota, Actinomycetota, and Acidobacteriota The literature data indicates that the Wood–Ljungdahl pathway has been described in acetogens, methanogens, and sulfate-reducing bacteria (Hügler and Sievert 2011), and more recently also in Actinomycetota, Bacillota, and Acidobacteriota (Vavourakis et al. 2018, Wong et al. 2018, Li et al. 2023). The predominance of these phyla in sub- and Maritime Antarctic mats (Fig. 1C) potentially contributes to the prevalence of the Wood–Ljungdahl pathway in these mats. Furthermore, the higher salinity in the deeper samples of the Transantarctic Mountains lakes could possibly explain the higher relative abundance of Bacteroidota, reflected in both the functional genes and rRNA genes, as conductivity has been positively correlated with the relative abundance of Bacteroidota in lakes in Patagonia, polar microbial mats and soils in the McMurdo Dry Valleys (Van Horn et al. 2013, Kim et al. 2016, Schiaffino et al. 2016, Tytgat et al. 2023).

Known differences in food web structure between regions are also reflected in variations in microbial community composition. rRNA gene sequences analysis (Fig. 1C) revealed a dominance of Pseudomonadota and Cyanobacteriota, consistent with an amplicon sequencing analysis of a larger set of lakes (Tytgat et al. 2023). Pseudomonadota were generally dominant in the Arctic, sub-Antarctic, and North–West Antarctic Peninsula lakes, which generally have vegetation in their catchments. By contrast, Cyanobacteriota were dominant in the North–East Antarctic Peninsula and Continental Antarctic mats with barren catchments. We hypothesize that these differences could be the result of differences in food web structure between regions. Indeed, Antarctic food webs are less complex compared to the Arctic due to the general lower diversity and the lack of large metazoans (Laybourn-Parry and Pearce 2007, Tytgat et al. 2023), resulting in a lower grazing rate and hence lower levels of bioturbation in Antarctic lakes, stimulating the abundance of Cyanobacteriota and Chlorophyta in benthic mats. By contrast, Arctic and sub-Antarctic lakes are generally dominated by grazing-tolerant ochrophytes and heterotrophic bacteria (Tytgat et al. 2023). Also in our study, heterotrophic bacteria were more abundant in the Arctic and sub-Antarctic samples and consistently also contributed considerably to many of the studied functional pathways. Pseudomonadota show a wide diversity of metabolic pathways and play important roles in global nutrient cycles (Madigan et al. 2010). This suggests Pseudomonadota are crucial players in the nutrient cycling within the benthic mats as was similarly proposed in other studies (Spain et al. 2009, Varin et al. 2010).

## Conclusions

The analysis of 17 microbial mat metagenomes from 15 lakes spread across the Arctic, sub-, Maritime, and Continental Antarctica, including the Transantarctic Mountains revealed a core set of functions involved in (cold) stress response and phosphorus cycling and metabolism in all the studied mats. This probably reflects the adaptation of the microbial mat communities to cold environments, as well as adaptation to oligotrophic conditions. Furthermore, this study highlights the considerable potential influence of environmental and climatic conditions, including temperature and sources and biochemical nature of organic matter input, on the functional genetic potential of microbial mat communities across polar lakes and how this potential could be shaped by community composition and food web structure. Overall, our findings emphasize the interplay between environmental conditions, microbial community composition, and functional genetic potential in shaping the evolution of biogeochemical processes in polar microbial mats.

## Supplementary Material

fiag060_Supplemental_Files

## Data Availability

The sequencing data generated in this study have been submitted to the European Nucleotide Archive (ENA) under the BioProject PRJEB59431 and accession numbers for the raw reads ERR10836078-ERR10836094.
