# Supplementary material for: Functional genetic potential of benthic microbial mat communities in Arctic, Antarctic, and sub-Antarctic lakes
Source: FEMS Microbiol Ecol. 2026 Jun 13;102(7):fiag060. doi: 10.1093/femsec/fiag060 (PMC13278500; doi:10.1093/femsec/fiag060)
Supplement: fiag060_Supplemental_Files [file fiag060_supplemental_files.zip › Supplementary data references supplementary table 2.docx]

**References supplementary table 2**

Acinas SG, Sánchez P, Salazar G et al. Metabolic Architecture of the Deep Ocean Microbiome. bioRxiv 2019, DOI: 10.1101/635680.

Beier S, Bertilsson S. Bacterial chitin degradation—mechanisms and ecophysiological strategies. Front Microbiol 2013;4:149.

Béjà O, Suzuki MT, Heidelberg JF et al. Unsuspected diversity among marine aerobic anoxygenic phototrophs. Nature 2002;415:630–3.

Berg IA, Kockelkorn D, Ramos-Vera WH et al. Autotrophic carbon fixation in archaea. Nat Rev Microbiol 2010;8:447–60.

Bergkemper F, Schöler A, Engel M et al. Phosphorus depletion in forest soils shapes bacterial communities towards phosphorus recycling systems. Environ Microbiol 2016;18:1988–2000.

Van Den Brink J, De Vries RP. Fungal enzyme sets for plant polysaccharide degradation. Applie Microbiology and Biotechnology 2011;91:1477–92.

Chen M, Zhang Y. Tracking the molecular evolution of photosynthesis through characterization of atomic contents of the photosynthetic units. Photosynth Res 2008;97:255–61.

Darwin AJ. The phage-shock-protein response. Mol Microbiol 2005;57:621–8.

Dyhrman ST. Microbial physiological ecology of the marine phosphorus cycle. In: Gasol JM, Kirchman DL (eds.), Microbial Ecology of the Oceans. 3rd ed. 2018, 377–434.

Dziewit L, Pyzik A, Romaniuk K et al. Novel molecular markers for the detection of methanogens and phylogenetic analyses of methanogenic communities. Front Microbiol 2015;6:694-undefined.

Gutiérrez-Preciado A, Saghaï A, Moreira D et al. Functional shifts in microbial mats recapitulate early Earth metabolic transitions. Nat Ecol Evol 2018;2:1700–8.

Hügler M, Sievert SM. Beyond the Calvin Cycle: Autotrophic carbon fixation in the ocean. Annu Rev Marine Sci 2011;3:261–89.

Imhoff JF, Rahn T, Künzel S et al. Phylogeny of Anoxygenic Photosynthesis Based on Sequences of Photosynthetic Reaction Center Proteins and a Key Enzyme in Bacteriochlorophyll Biosynthesis, the Chlorophyllide Reductase. Microorganisms 2019;7:576.

Imlay JA. The molecular mechanisms and physiological consequences of oxidative stress: lessons from a model bacterium. Nat Rev Microbiol 2013;11:443–54.

Janusz G, Pawlik A, Sulej J et al. Lignin degradation: microorganisms, enzymes involved, genomes analysis and evolution. FEMS Microbiol Rev 2017;41:941–62.

Joly N, Engl C, Jovanovic G et al. Managing membrane stress: the phage shock protein (Psp) response, from molecular mechanisms to physiology. FEMS Microbiol Rev 2010;34:797–827.

Kamp A, Høgslund S, Risgaard-Petersen N et al. Nitrate Storage and Dissimilatory Nitrate Reduction by Eukaryotic Microbes. Front Microbiol 2015;6:1492.

Kasalický V, Zeng Y, Piwosz K et al. Aerobic anoxygenic photosynthesis is commonly present within the genus Limnohabitans. Appl Environ Microbiol 2018;84, DOI: 10.1128/AEM.02116-17.

Kindler GS, Wong HL, Larkum AWD et al. Metagenomic insights into ecosystem function in the microbial mats of Blue Holes, Shark Bay. bioRxiv 2020, DOI: 10.1101/2020.09.18.304444.

Knittel K, Boetius A. Anaerobic Oxidation of Methane: Progress with an Unknown Process. Annu Rev Microbiol 2009;63:311–34.

Koo H, Hakim JA, Morrow CD et al. Metagenomic Analysis of Microbial Community Compositions and Cold-Responsive Stress Genes in Selected Antarctic Lacustrine and Soil Ecosystems. Life 2018;8:29.

Krämer R. Bacterial stimulus perception and signal transduction: Response to osmotic stress. The Chemical Record 2010;10:217–29.

McDonald IR, Bodrossy L, Chen Y et al. Molecular ecology techniques for the study of aerobic methanotrophs. Appl Environ Microbiol 2008;74:1305–15.

Nelson N, Ben-Shem A. The complex architecture of oxygenic photosynthesis. Nat Rev Mol Cell Biol 2004;5:971–82.

Pérez J, Munõz-Dorado J, De La Rubia T et al. Biodegradation and biological treatments of cellulose, hemicellulose and lignin: an overview. International Microbiology 2002;5:53–63.

Rodrigues DF, Tiedje JM. Coping with Our Cold Planet. Appl Environ Microbiol 2008;74:1677–86.

Ruiz-Fernández P, Ramírez-Flandes S, Rodríguez-León E et al. Autotrophic carbon fixation pathways along the redox gradient in oxygen-depleted oceanic waters. Environ Microbiol Rep 2020;12:334–41.

Salazar G, Paoli L, Alberti A et al. Gene Expression Changes and Community Turnover Differentially Shape the Global Ocean Metatranscriptome. Cell 2019;179:1083.

Santos PC Dos, Fang Z, Mason SW et al. Distribution of nitrogen fixation and nitrogenase-like sequences amongst microbial genomes. BMC Genomics 2012;13:162-undefined.

Smith GJ, Wrighton KC. Metagenomic Approaches Unearth Methanotroph Phylogenetic and Metabolic Diversity. Curr Issues Mol Biol 2019;33:57–84.

Sunagawa S, Coelho LP, Chaffron S et al. Structure and function of the global ocean microbiome. Science (1979) 2015;348, DOI: 10.1126/SCIENCE.1261359/SUPPL_FILE/SUNAGAWA_TABLES1.XLSX.

Varin T, Lovejoy C, Jungblut AD et al. Metagenomic analysis of stress genes in microbial mat communities from Antarctica and the high Arctic. Appl Environ Microbiol 2012;78:549–59.

Wong HL, White RA, Visscher PT et al. Disentangling the drivers of functional complexity at the metagenomic level in Shark Bay microbial mat microbiomes. ISME J 2018;12:2619–39.
